# Serum Tetranectin Level and Its Association with Metabolic Parameters in Women with Gestational Diabetes Mellitus: A Case–Control Study

**DOI:** 10.3390/healthcare14172775

**Published:** 2026-09-01

**Authors:** Ozgur Ozdemir, Durmus Onder, Yasin Karadag, Ahmet Ilker Eryilmaz

**Affiliations:** 1 Department of Obstetrics and Gynecology, University of Health Sciences, Antalya Education and Research Hospital, 07100 Antalya, Turkey; 2 Department of Obstetrics and Gynecology, Mardin Kızıltepe State Hospital, 47400 Mardin, Turkey; dr.yasinkaradag@gmail.com; 3 Department of Obstetrics and Gynecology, Antalya City Hospital, 07100 Antalya, Turkey

**Keywords:** gestational diabetes mellitus, tetranectin, CLEC3B, biomarker, insulin resistance, pregnancy

## Abstract

Background/Objectives: Gestational diabetes mellitus (GDM) is a heterogeneous metabolic disorder characterized by impaired glucose regulation and insulin resistance. Tetranectin is a circulating protein implicated in metabolic regulation and diabetes-related dysfunction; however, serum tetranectin levels have not previously been investigated in women with GDM. This study aimed to compare serum tetranectin levels between women with GDM and normoglycemic pregnant controls and to evaluate their associations with metabolic parameters and discriminatory performance for GDM. Methods: This prospective case–control study included 89 pregnant women recruited at the Department of Obstetrics and Gynecology, Antalya Education and Research Hospital, between January 2023 and March 2025, comprising 45 women with GDM and 44 normoglycemic controls. GDM was diagnosed using a 75 g oral glucose tolerance test. Serum tetranectin levels were measured using a commercially available enzyme-linked immunosorbent assay. Clinical, obstetric, metabolic, and laboratory parameters were compared between groups. Correlations between serum tetranectin and metabolic parameters were assessed using Spearman’s correlation analysis. Binary logistic regression was performed to evaluate the independent association between serum tetranectin and GDM. Receiver operating characteristic curve analysis was used to assess discriminatory performance. Results: No statistically significant differences were observed in baseline maternal and obstetric characteristics between the groups. Serum tetranectin levels were significantly higher in women with GDM than in normoglycemic controls (155.42 [147.99–181.36] ng/mL vs. 103.82 [66.89–159.76] ng/mL, *p* < 0.001). Serum tetranectin was positively correlated with HbA1c (ρ = 0.431, *p* < 0.001), fasting glucose (ρ = 0.350, *p* < 0.001), 2 h OGTT glucose (ρ = 0.277, *p* = 0.009), body weight (ρ = 0.270, *p* = 0.011), HOMA-IR (ρ = 0.246, *p* = 0.024), triglyceride levels (ρ = 0.243, *p* = 0.047), diastolic blood pressure (ρ = 0.241, *p* = 0.049), and body mass index (ρ = 0.224, *p* = 0.034). After Bonferroni correction for multiple comparisons, only the correlations with HbA1c and fasting glucose remained statistically significant. In binary logistic regression analysis, each 10 ng/mL increase in serum tetranectin was associated with higher odds of GDM in both crude and adjusted models. After adjustment for maternal age, gestational age at sampling, and body mass index, serum tetranectin remained independently associated with GDM (OR: 1.17, 95% CI: 1.06–1.28, *p* = 0.001). Receiver operating characteristic analysis demonstrated moderate discriminatory performance, with an AUC of 0.747 (95% CI: 0.635–0.860, *p* < 0.001). The optimal cut-off value was 124.59 ng/mL, yielding 95.6% sensitivity (95% CI: 84.9–99.5%) and 63.6% specificity (95% CI: 47.8–77.6%). Conclusions: Serum tetranectin levels were significantly elevated in women with GDM and remained independently associated with GDM status after adjustment for maternal age, gestational age at sampling, and body mass index. Serum tetranectin showed associations with several metabolic parameters; however, after multiple-testing correction, only fasting glucose and HbA1c remained significantly correlated with TN. Although its discriminatory performance was moderate, serum tetranectin may represent a biologically plausible circulating biomarker associated with the metabolic environment of GDM. Larger longitudinal studies are needed to validate these findings and clarify the mechanistic role of tetranectin in GDM.

## 1. Introduction

Gestational diabetes mellitus (GDM) is one of the most common metabolic disorders of pregnancy and is characterized by glucose intolerance that is first recognized during gestation. Its prevalence varies according to population and diagnostic criteria, and approximately 5.4% of pregnancies in Europe are reported to be affected by GDM [1]. Its pathophysiology is closely related to the progressive insulin resistance of pregnancy, which is driven by placental hormones, maternal adiposity, and metabolic stress. Although this physiological insulin-resistant state is normally compensated by increased pancreatic β-cell function, inadequate β-cell adaptation results in maternal hyperglycemia and the clinical development of GDM [1]. GDM is associated with clinically important adverse outcomes, including fetal macrosomia, preterm delivery, fetal and neonatal complications, and increased perinatal morbidity and mortality. In the long term, it is also associated with an increased risk of type 2 diabetes and cardiovascular disease in mothers and metabolic disorders in offspring [1].

The current diagnosis of GDM is mainly based on oral glucose tolerance testing. Although the OGTT remains the standard diagnostic approach, it reflects glycemic responses during a single testing session and provides limited information regarding the broader metabolic heterogeneity and biological pathways underlying GDM [1,2]. GDM extends beyond hyperglycemia and involves alterations in insulin resistance, lipid metabolism, inflammatory pathways, endothelial function, fibrinolysis, and extracellular matrix remodeling. Therefore, there is increasing interest in identifying circulating biomarkers that may provide biological information beyond conventional glycemic parameters. In this context, assessment of tetranectin may complement, rather than replace, OGTT-based diagnosis by providing additional information on the metabolic disturbances associated with GDM. Recent metabolomic and proteomic studies have similarly suggested that circulating biomarkers may contribute to a more comprehensive characterization of the GDM phenotype [1,2].

Tetranectin (TN), also known as C-type lectin domain family 3 member B (CLEC3B), is a plasminogen-binding C-type lectin involved in fibrinolysis and tissue remodeling [3,4]. Although circulating TN has been investigated as a biomarker in several disease states [4,5], the available metabolic evidence has largely been derived from experimental models and non-gestational diabetes-related conditions rather than GDM. Its potential relevance to GDM is supported indirectly by evidence linking TN to glucose-responsive adipose secretion, pancreatic β-cell function, and diabetes-associated metabolic and endothelial dysfunction [6,7,8]. Thus, these previous findings provide a biological rationale for studying TN in GDM but should not be interpreted as direct evidence of its role in gestational diabetes.

**Figure 1:**
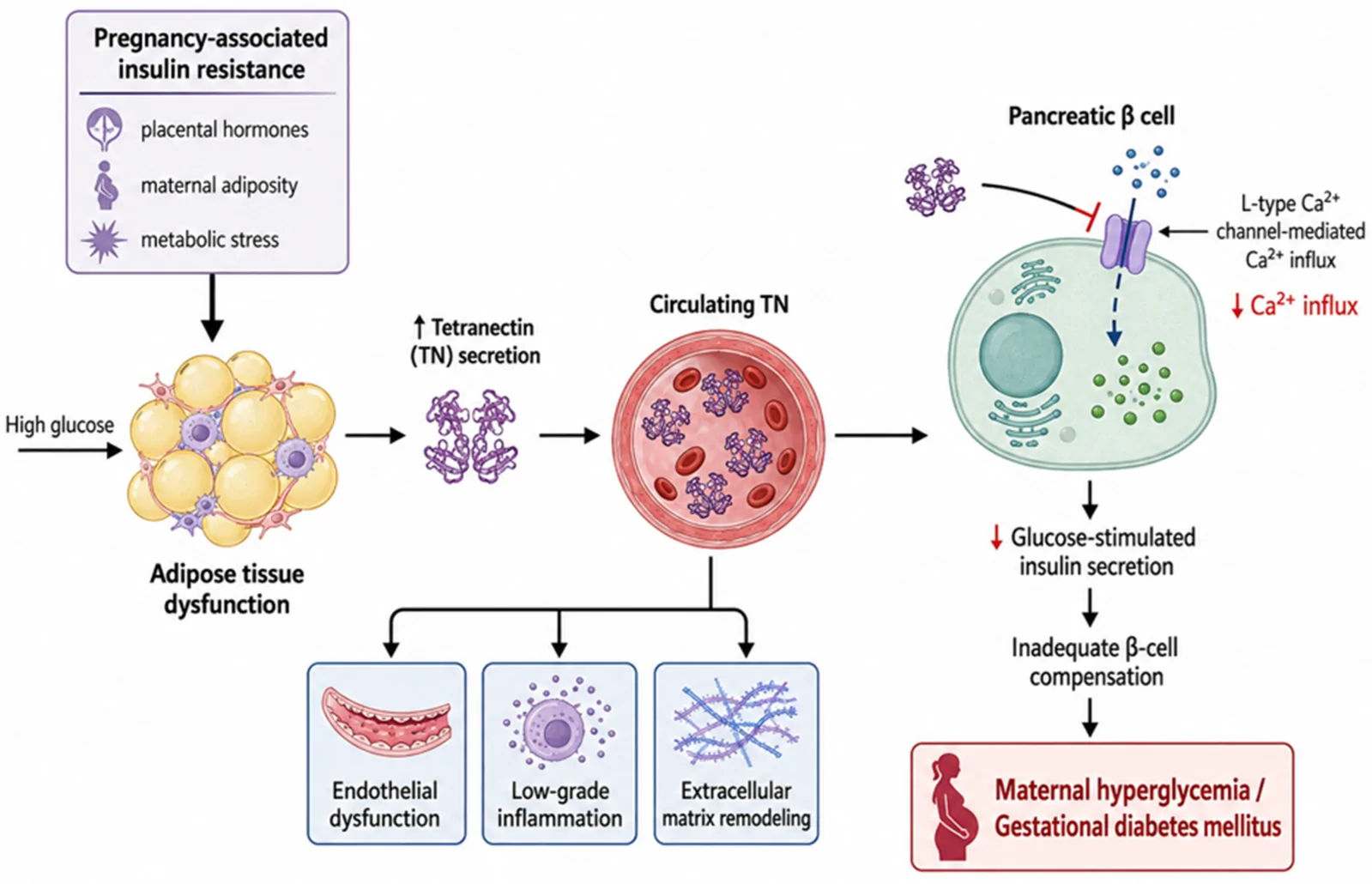
Proposed role of tetranectin in the adipocyte–β-cell axis and its potential relevance to gestational diabetes mellitus. High-glucose conditions and adipose tissue dysfunction may increase tetranectin secretion from adipocytes. Circulating tetranectin may then interact with pancreatic β cells and suppress glucose-stimulated insulin secretion by inhibiting L-type calcium channel-mediated calcium influx. In the setting of pregnancy-induced insulin resistance, this may contribute to inadequate β-cell compensation and maternal hyperglycemia. Tetranectin may also be linked to endothelial dysfunction, inflammation, and extracellular matrix remodeling, thereby reflecting the broader metabolic environment of GDM. This figure represents a proposed biological model based on experimental and observational evidence and does not imply proven causality in GDM.

Experimental evidence further supports a possible role of TN in glucose homeostasis. Liu et al. identified TN as an adipocyte-enriched secretory protein whose expression is stimulated by high glucose and whose circulating levels are increased in diabetes. Mechanistically, TN suppressed glucose-stimulated insulin secretion from pancreatic β cells by inhibiting L-type calcium channel-mediated calcium influx, thereby linking adipose tissue dysfunction with impaired β-cell function [6]. In addition, TN has been detected in pancreatic islet cells and has been implicated in pericellular proteolysis and tissue remodeling processes relevant to islet biology [7]. These findings suggest that TN may participate in adipose tissue–β-cell crosstalk, a mechanism highly relevant to GDM, in which pregnancy-induced insulin resistance requires adequate β-cell compensation. The proposed mechanistic framework is summarized in Figure 1.

TN may also be relevant to the vascular and inflammatory components of metabolic disease. In children with type 1 diabetes mellitus, elevated circulating TN levels have been reported together with oxidative stress and endothelial/platelet activation markers [8]. Moreover, exercise training in overweight men with features of metabolic syndrome was associated with increased skeletal muscle and plasma TN levels, suggesting that TN may be involved in systemic fibrinolysis and vascular homeostasis [9]. Given that GDM is associated with insulin resistance, adipose tissue dysfunction, low-grade inflammation, endothelial activation, and altered extracellular matrix remodeling, TN represents a biologically plausible candidate biomarker in this setting.

Several adipokines, particularly adiponectin and leptin, have previously been investigated in GDM, with lower adiponectin and higher leptin levels repeatedly reported in women who develop GDM [10]. TN was selected as a candidate biomarker not because of an assumed advantage over these established adipokines but because it represents a mechanistically distinct and previously unexplored pathway in GDM. Unlike the extensively studied adiponectin and leptin pathways, experimental evidence directly links TN to glucose-responsive adipocyte secretion and suppression of glucose-stimulated insulin secretion from pancreatic β cells [6]. Thus, TN may provide complementary information regarding adipose tissue–β-cell crosstalk rather than serving as a substitute for existing biomarkers or OGTT-based diagnosis.

To date, no study has specifically investigated the relationship between serum TN levels and GDM. We hypothesized that serum TN levels would be elevated in women with GDM and would be associated with metabolic parameters reflecting glucose intolerance and insulin resistance. Therefore, this study aimed to compare serum TN levels between women with GDM and normoglycemic pregnant controls and to evaluate their associations with clinical and metabolic parameters. In addition, we assessed the discriminatory performance of serum TN to determine whether it could provide clinically relevant complementary information for distinguishing GDM from normoglycemic pregnancies. This represents a novel exploratory assessment of the potential diagnostic utility of TN in GDM.

## 2. Materials and Methods

### 2.1. Study Design and Patients

This study was designed as a prospective case–control study. Pregnant women who were followed at the Department of Obstetrics and Gynecology at Antalya Education and Research Hospital between January 2023 and March 2025 were evaluated for eligibility. Pregnant women attending routine antenatal follow-up during the study period were consecutively evaluated for eligibility based on predefined inclusion and exclusion criteria. The study protocol was approved by the Institutional Ethics Committee of Antalya Education and Research Hospital (approval date: 14 October 2021; approval number: 16/7). This ethical approval remained valid throughout the recruitment and study period, and no renewal or additional approval was required. All participants provided written informed consent before enrollment, and the study was conducted in accordance with the principles of the Declaration of Helsinki.

Pregnant women aged 18–45 years who were between 24 and 28 weeks of gestation and attended routine antenatal follow-up were screened for inclusion. Gestational age was calculated according to the last menstrual period and confirmed by first-trimester ultrasonographic measurements. Eligible participants were classified into two groups based on the results of the 75 g oral glucose tolerance test. The GDM group consisted of pregnant women diagnosed with gestational diabetes mellitus, whereas the control group included normoglycemic pregnant women with normal 75 g oral glucose tolerance test results and no known systemic, metabolic, inflammatory, or endocrine disease.

Women were excluded if they had pre-existing type 1 or type 2 diabetes mellitus, multiple pregnancy, chronic hypertension, preeclampsia, renal, hepatic, cardiovascular, autoimmune, inflammatory, infectious, thyroid, or other endocrine disorders. Active smoking, alcohol or substance use, use of corticosteroids, anti-inflammatory drugs or medications affecting glucose metabolism, fetal anomaly, intrauterine growth restriction, assisted reproductive technology pregnancy, and incomplete clinical or laboratory data were also considered exclusion criteria.

### 2.2. Diagnosis of Gestational Diabetes Mellitus

GDM was diagnosed using a 75 g oral glucose tolerance test performed between 24 and 28 weeks of gestation, according to the International Association of Diabetes and Pregnancy Study Groups criteria [11]. After an overnight fast of at least 8 h, venous blood samples were obtained to measure fasting plasma glucose. Subsequently, participants ingested 75 g of oral glucose, and plasma glucose levels were measured at 1 and 2 h post-ingestion. GDM was diagnosed when one or more of the following plasma glucose thresholds were met or exceeded: fasting plasma glucose ≥ 92 mg/dL, 1 h plasma glucose ≥ 180 mg/dL, or 2 h plasma glucose ≥ 153 mg/dL. Pregnant women with glucose values below all diagnostic thresholds were considered to have normal glucose tolerance and were included in the control group.

### 2.3. Clinical and Demographic Data Collection

The following clinical, obstetric, and laboratory variables were recorded for each participant: maternal age, gestational age at sampling, gravida, parity, abortus, current body mass index, gestational weight gain, systolic and diastolic blood pressure, fasting plasma glucose, 1 h and 2 h OGTT glucose values, HbA1c, fasting insulin, lipid profile, and routine biochemical parameters. Current body mass index was calculated as weight in kilograms divided by height in meters squared using body weight measured at study enrollment (24–28 weeks of gestation). Current BMI was used because it was assessed contemporaneously with serum TN and the metabolic measurements, and therefore reflects maternal adiposity at the time of biomarker evaluation. Gestational weight gain was evaluated separately and was calculated as the difference between body weight recorded at the beginning of pregnancy and body weight measured at study enrollment.

### 2.4. Blood Sample Collection and Measurement of Serum TN Levels

Venous blood samples were obtained from all participants after an overnight fast of at least 8 h. Blood samples were collected in the morning, before the oral glucose tolerance test, to minimize potential diurnal variation and preanalytical variability. For serum TN measurement, blood samples were collected into serum separator tubes and allowed to clot at room temperature for 10–20 min. The samples were then centrifuged at 2000–3000 rpm for 20 min, and the separated serum was carefully collected without disturbing the sediment. Serum TN measurements were performed after participant recruitment was completed. Depending on the date of sample collection, storage duration varied; the earliest samples were stored for approximately 26 months before analysis. All aliquots were continuously maintained at −80 °C under identical storage conditions, and repeated freeze–thaw cycles were avoided. All study samples were subsequently analyzed in a single analytical batch to minimize inter-assay variability. Serum TN levels were measured using a commercially available sandwich enzyme-linked immunosorbent assay kit for human tetranectin/CLEC3B (Human Tetranectin/CLEC3B ELISA Kit, BT LAB, Cat. No. E6262Hu, Shanghai, China). The assay is based on a pre-coated microplate with human CLEC3B antibody. Tetranectin/CLEC3B present in the serum sample binds to the immobilized antibody, followed by the addition of biotinylated human CLEC3B antibody and streptavidin-HRP. After washing, substrate solutions are added, and the color intensity develops in proportion to the amount of tetranectin/CLEC3B in the sample. The reaction is stopped with a stop solution, and the optical density is measured at 450 nm using a microplate reader. The assay standard curve ranged from 2 to 600 ng/mL, and the analytical sensitivity was 1.23 ng/mL. Samples with optical density values exceeding the upper limit of the standard curve were diluted using the assay diluent and re-assayed according to the laboratory protocol for out-of-range ELISA measurements. The final TN concentrations were calculated after correction for the dilution factor. This procedure was applied only to samples whose initial measurements exceeded the upper limit of the calibration curve. Except for the laboratory-defined dilution procedure for out-of-range samples, all other assay steps were performed in accordance with the manufacturer’s protocol. The reported intra-assay coefficient of variation ranged from 4.52% to 5.57%, and the inter-assay coefficient of variation was <10%. Samples and standards were analyzed in duplicate. A coefficient of variation of <10% between duplicate measurements was considered acceptable, and the mean of the two measurements was used for statistical analysis. Laboratory personnel were blinded to participants’ clinical group allocation.

### 2.5. Statistical Analysis

Statistical analyses were performed using IBM SPSS Statistics for Windows, version 32.0.0 (IBM Corp., Armonk, NY, USA). The normality of continuous variables was assessed separately in the GDM and control groups using the Shapiro–Wilk test, along with histograms and Q-Q plots. Continuous variables with normal distribution were presented as mean ± standard deviation, whereas non-normally distributed variables were presented as median [interquartile range]. Categorical variables were expressed as numbers and percentages. Comparisons between the GDM and control groups were performed using the independent-samples *t*-test for normally distributed variables and the Mann–Whitney U test for non-normally distributed variables. Categorical variables were compared using the chi-square test or Fisher’s exact test, as appropriate. Because serum TN levels were not normally distributed, correlations between serum TN levels and clinical, metabolic, and laboratory parameters were analyzed using Spearman’s rank correlation test. To account for multiple comparisons, a Bonferroni correction was applied across the 34 correlation analyses, resulting in an adjusted significance threshold of *p* < 0.00147. Binary logistic regression analysis was performed to evaluate whether serum TN level was independently associated with GDM. Gestational diabetes mellitus status was entered as the dependent variable. Serum TN was entered into the model as a continuous variable, expressed per 10-ng/mL increase. The adjusted model included maternal age, gestational age at sampling, and body mass index as prespecified clinically relevant potential confounders. Maternal age was selected because of its established association with GDM risk; gestational age at sampling was included to account for gestation-related metabolic and hormonal variation; and BMI was included because maternal adiposity is associated with GDM and may also influence TN biology. Glycemic and insulin-resistance variables, including OGTT glucose values, HbA1c, insulin, and HOMA-IR, were not included as covariates because they are closely related to the diagnostic definition or metabolic phenotype of GDM, and their inclusion could lead to overadjustment. Given the number of GDM cases, the model was intentionally kept parsimonious to limit overfitting. Odds ratios and 95% confidence intervals were calculated. Receiver operating characteristic curve analysis was performed to evaluate the discriminatory performance of serum TN levels for differentiating women with GDM from normoglycemic pregnant controls. The area under the curve, 95% confidence interval, optimal cut-off value, sensitivity, specificity, positive predictive value, negative predictive value, and accuracy were calculated. Because positive and negative predictive values are prevalence-dependent, these measures were considered sample-specific descriptive estimates and were interpreted cautiously in the context of the case–control design. Exact binomial 95% confidence intervals were calculated for sensitivity and specificity. The optimal cut-off value was determined using the Youden index.

No a priori sample-size calculation was performed because no prior data were available on serum TN levels in women with GDM to provide a reliable estimate of effect size. The final sample size was therefore determined by the number of eligible participants recruited during the predefined study period. Post hoc power analysis was performed for the primary comparison of serum TN levels between the study groups. A two-sided *p*-value of <0.05 was considered statistically significant.

## 3. Results

### 3.1. Baseline Maternal and Obstetric Characteristics

**Figure 2:**
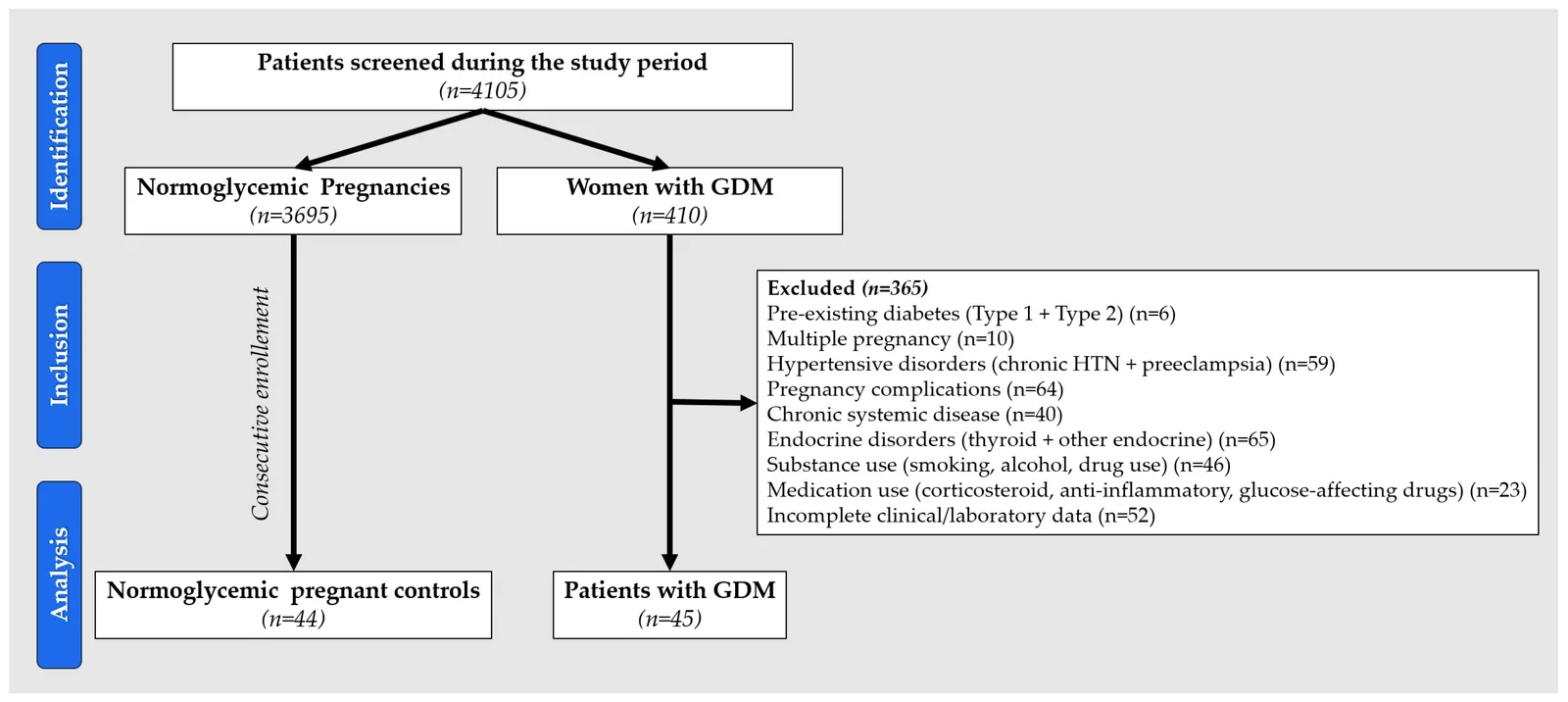
Participant flow diagram showing screening, exclusions, and final inclusion of women with gestational diabetes mellitus and normoglycemic pregnant controls.

During the study period, 4105 pregnant women were screened. Of these, 3695 had normoglycemic pregnancies, and 410 were evaluated for inclusion in the GDM group. Among the latter, 365 were excluded because of pre-existing diabetes (*n* = 6), multiple pregnancy (*n* = 10), hypertensive disorders including chronic hypertension or preeclampsia (*n* = 59), other pregnancy complications (*n* = 64), chronic systemic disease (*n* = 40), thyroid or other endocrine disorders (*n* = 65), smoking, alcohol, or substance use (*n* = 46), use of corticosteroids, anti-inflammatory drugs, or medications affecting glucose metabolism (*n* = 23), or incomplete clinical or laboratory data (*n* = 52). This resulted in 45 eligible women with GDM. Forty-four eligible normoglycemic pregnant women were consecutively enrolled as controls, resulting in a final study population of 89 participants. Participant selection and study flow are summarized in Figure 2.

The baseline maternal and obstetric characteristics of the study groups are presented in Table 1. Between-group differences were evaluated using effect estimates with 95% confidence intervals in addition to conventional hypothesis testing. No statistically significant differences were observed for the baseline variables examined; however, the magnitude and precision of individual between-group differences are presented in Table 1 rather than interpreting non-significant *p*-values as evidence of equivalence. Although maternal age did not differ significantly between groups (30.4 ± 6.2 vs. 28.0 ± 5.4 years, *p* = 0.086), the approximately 2.4-year difference was considered potentially clinically relevant; therefore, maternal age was included as a confounder in the adjusted logistic regression models.

### 3.2. Glucose Metabolism and Laboratory Parameters

Glucose metabolism and laboratory parameters are summarized in Table 2. As expected, OGTT fasting glucose, 1 h glucose, and 2 h glucose values were significantly higher in the GDM group compared with the control group. Similarly, insulin levels, recalculated HOMA-IR values, and HbA1c levels were significantly higher in women with GDM. In contrast, there were no significant differences between the groups in hemoglobin, hematocrit, white blood cell count, platelet count, total protein, albumin, triglycerides, LDL cholesterol, HDL cholesterol, VLDL cholesterol, blood urea nitrogen, total cholesterol, AST, ALT, LDH, or creatinine levels.

### 3.3. Serum TN Levels

**Figure 3:**
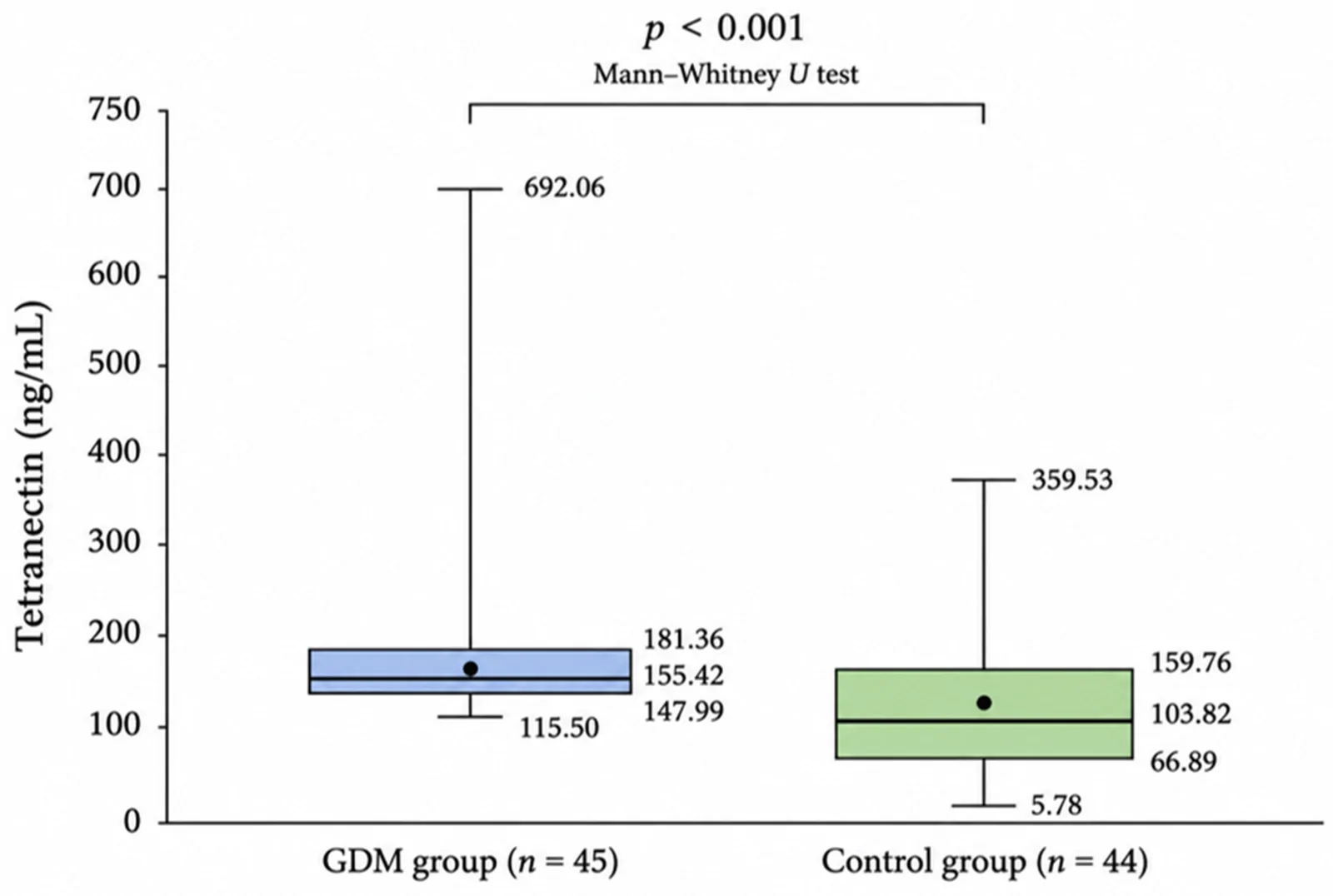
Distribution of serum TN levels in women with GDM and normoglycemic pregnant controls. Box plots show serum TN levels in the GDM group (*n* = 45) and the control group (*n* = 44). The horizontal line within each box represents the median, boxes indicate the interquartile range, whiskers represent the minimum and maximum values, and black dots indicate the mean values. Serum TN levels were significantly higher in the GDM group than in the control group (Mann–Whitney U test, *p* < 0.001). GDM: gestational diabetes mellitus.

Serum TN levels were significantly higher in women with GDM than in normoglycemic pregnant controls. The median serum TN level was 155.42 [147.99–181.36] ng/mL in the GDM group and 103.82 [66.89–159.76] ng/mL in the control group (*p* < 0.001). Additional descriptive analysis showed that the mean serum TN level was 175.91 ± 84.57 ng/mL in the GDM group and 116.20 ± 75.49 ng/mL in the control group. The distribution of serum TN levels in the study groups is shown in Figure 3.

### 3.4. Correlation Analysis

The correlation analysis between serum TN levels and clinical, metabolic, and biochemical parameters is shown in Table 3. Serum TN levels showed nominal positive correlations with diastolic blood pressure, weight, body mass index, OGTT fasting glucose, OGTT 2 h glucose, HOMA-IR, HbA1c, and triglyceride levels. After Bonferroni correction for multiple comparisons (adjusted significance threshold, *p* < 0.00147), only the correlations with fasting glucose (ρ = 0.350, *p* < 0.001) and HbA1c (ρ = 0.431, *p* < 0.001) remained statistically significant. HbA1c showed the strongest correlation with serum TN.

### 3.5. Binary Logistic Regression Analysis

Serum TN was independently associated with GDM after adjustment for maternal age, gestational age at sampling, and body mass index. Each 10-ng/mL increase in serum TN was associated with a 17% increase in the odds of GDM (adjusted OR: 1.17, 95% CI: 1.06–1.28, *p* = 0.001; Table 4).

### 3.6. ROC Analysis

**Figure 4:**
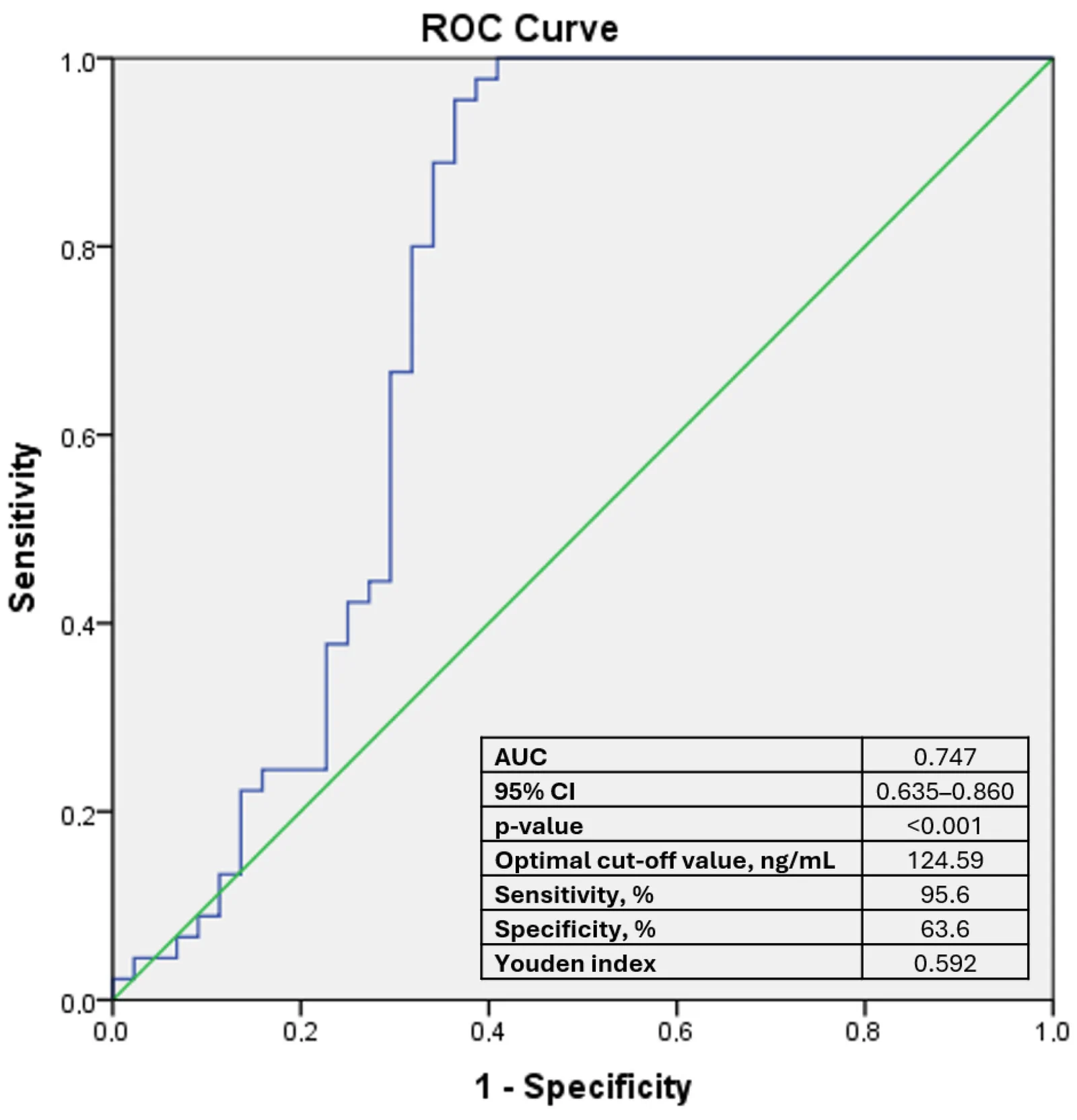
Receiver operating characteristic curve of serum TN for discriminating GDM from normoglycemic pregnancy.

ROC analysis was performed to evaluate the discriminatory performance of serum TN for differentiating women with GDM from normoglycemic pregnant controls. Serum TN demonstrated moderate discriminatory performance, with an AUC of 0.747 (95% CI: 0.635–0.860, *p* < 0.001). The optimal cut-off value was 124.59 ng/mL. At this threshold, serum TN showed a sensitivity of 95.6% (95% CI: 84.9–99.5%) and a specificity of 63.6% (95% CI: 47.8–77.6%). The Youden index was 0.592, with a positive predictive value of 72.9%, a negative predictive value of 93.3%, and an overall accuracy of 79.8%. The ROC curve is shown in Figure 4.

### 3.7. Post Hoc Power Analysis

A post hoc power analysis was performed for the primary comparison of serum TN levels between the GDM and control groups. Although the primary comparison was performed using the Mann–Whitney U test because serum TN levels were not normally distributed, the standardized mean difference was also calculated as an additional effect size estimate. Based on the observed mean serum TN levels, Cohen’s d was 0.74. With the final sample size of 45 women with GDM and 44 normoglycemic controls and a two-sided alpha level of 0.05, the achieved statistical power was 93.48%.

## 4. Discussion

In this prospective case–control study, serum TN levels were significantly higher in women with GDM than in normoglycemic pregnant controls. To the best of our knowledge, this is the first study to evaluate circulating TN levels specifically in women with GDM. In addition to the between-group difference, serum TN was correlated with several metabolic parameters; however, after multiple-testing correction, only the associations with fasting glucose and HbA1c remained statistically significant. Moreover, the association between TN and GDM persisted after adjustment for maternal age, gestational age at sampling, and BMI. These findings suggest that TN is not merely elevated in GDM but may also be linked to the broader metabolic environment characteristic of this condition. Although ROC analysis demonstrated only moderate discriminatory performance, the overall results support TN as a biologically plausible circulating biomarker associated with GDM.

GDM is a complex metabolic disorder that extends beyond isolated hyperglycemia and is characterized by progressive insulin resistance together with insufficient pancreatic β-cell compensation [1,2]. The observed increase in serum TN levels in women with GDM may reflect altered metabolic adaptation and underlying adipose tissue dysfunction. TN, encoded by the CLEC3B gene, is a plasminogen-binding C-type lectin traditionally associated with fibrinolysis, proteolytic regulation, tissue remodeling, and extracellular matrix-related biological processes [3,4]. Experimental studies have also linked TN to tissue repair and remodeling, as delayed tendon healing has been demonstrated in tetranectin-deficient mice, and increased TN levels have been reported in pathological tissue environments characterized by extracellular matrix remodeling and inflammation [12,13]. More recently, TN has been identified as an adipocyte-enriched secretory protein, with evidence indicating it is abundant in white adipose tissue and may function as an adipokine involved in metabolic regulation [6,14]. Experimental data indicate that high glucose levels may stimulate TN expression and secretion from mature adipocytes. At the molecular level, high-glucose conditions have been shown to activate the p38 MAPK/TXNIP/thioredoxin/OCT4 signaling pathway in adipose tissue, providing a potential mechanism for increased TN synthesis and release into the circulation [6]. Once in the bloodstream, TN may participate in adipose tissue–β-cell crosstalk by binding to pancreatic β cells with high selectivity [6,7].

Mechanistically, TN has been shown to suppress glucose-stimulated insulin secretion by inhibiting L-type calcium channel-mediated calcium influx in pancreatic β cells [6]. In the context of GDM, where pregnancy-induced insulin resistance requires an adequate compensatory increase in β-cell insulin secretion, this TN-mediated suppression of insulin release may contribute to impaired metabolic adaptation. Furthermore, previous diabetes-related studies have linked elevated TN levels to oxidative stress markers, such as lipid hydroperoxides, and to indicators of endothelial and platelet activation, suggesting that TN may also be associated with the vascular and inflammatory components of diabetes-related metabolic dysfunction [8]. While these biological mechanisms support TN as a relevant circulating marker of the metabolic environment in GDM, the observational nature of the present study precludes a definitive determination of whether increased TN is a causal mediator or a secondary response to the metabolic disturbances associated with the disease.

Serum TN levels were positively correlated with several indices of maternal metabolic dysfunction, including fasting glucose, 2 h OGTT glucose, HOMA-IR, HbA1c, BMI, body weight, triglyceride levels, and diastolic blood pressure. However, after Bonferroni correction for multiple comparisons, only the correlations with fasting glucose and HbA1c remained statistically significant, with the strongest association observed for HbA1c. These findings should be interpreted cautiously because the study groups were defined according to glucose tolerance status. In particular, fasting glucose is part of the diagnostic criteria for GDM, and therefore its correlation with TN may partly reflect the glycemic separation between the GDM and control groups rather than an independent association. Although HbA1c was not used to define GDM status, its relationship with TN may likewise be influenced by the overall differences in glycemic status between the groups. GDM is characterized not only by abnormal OGTT values but also by broader disturbances in glucose metabolism, insulin resistance, lipid metabolism, and inflammatory pathways [1,2]. Previous pregnancy-related proteomic data have shown that an oral glucose load can induce changes in circulating proteins related to inflammation, oxidative stress, adipogenesis, and advanced glycation-related pathways during mid-pregnancy [15]. In addition, studies of diabetic pregnancy have shown that indices of glycemic burden, including HbA1c and continuous glucose monitoring-derived parameters, are clinically relevant for predicting adverse fetal growth outcomes [16]. Accordingly, the present correlations should be regarded as exploratory evidence linking TN to the glycemic environment of GDM rather than as evidence that TN independently reflects glycemic burden. The nominal associations with 2 h OGTT glucose, HOMA-IR, BMI, body weight, and triglyceride levels were not statistically significant after multiple-testing correction and therefore should not be overinterpreted. Nevertheless, these findings remain biologically compatible with experimental evidence indicating that TN is an adipocyte-enriched secretory protein involved in glucose and lipid homeostasis [6,14]. Overall, serum TN should be considered a complementary exploratory marker of the broader metabolic milieu in GDM, rather than a direct substitute for established glycemic or insulin resistance parameters.

ROC analysis showed that serum TN had moderate discriminatory performance for differentiating women with GDM from normoglycemic pregnant controls. The AUC of 0.747 indicates measurable discriminative ability; however, this level of performance is insufficient to support TN as a stand-alone diagnostic test. At the optimal cut-off value of 124.59 ng/mL, TN showed high sensitivity but modest specificity; the observed predictive values should be interpreted cautiously because they depend on the case–control sampling distribution. This pattern suggests that TN may be more useful as an adjunctive or exploratory biomarker rather than as a replacement for OGTT-based diagnosis, which remains the standard approach for identifying clinically relevant hyperglycemia in pregnancy [11,17]. The moderate AUC also indicates that TN alone does not capture the full biological heterogeneity of GDM. This is consistent with current evidence suggesting that GDM involves multiple metabolic, inflammatory, lipid-related, and proteomic pathways, and that single biomarkers are unlikely to fully represent this complex phenotype [1,2]. Therefore, future studies should evaluate whether TN improves risk stratification when combined with clinical variables, glycemic indices, inflammatory markers, or other proteomic biomarkers in multimarker prediction models. Such models should be tested in larger independent cohorts and reported in accordance with diagnostic accuracy standards before any clinical application can be considered [18].

This study has several strengths. First, it was designed as a prospective case–control study and included clearly defined GDM and normoglycemic control groups evaluated during the standard 24–28-week screening period. Second, baseline maternal and obstetric characteristics were carefully assessed, with effect estimates and 95% confidence intervals reported for between-group differences and clinically relevant potential confounders considered in the adjusted analysis. Third, blood samples were obtained under standardized fasting conditions prior to the OGTT, and serum TN levels were measured using a commercially available ELISA kit. Samples were analyzed in duplicate, and laboratory personnel were blinded to clinical group allocation. In addition, the association between serum TN and GDM was evaluated not only by between-group comparison but also by correlation analysis, multivariable logistic regression adjusted for maternal age, gestational age at sampling, and BMI, and ROC analysis. However, several limitations should also be acknowledged. This was a single-center study with a relatively small sample, and the findings require external validation in larger, more diverse populations. Because of the case–control design, causal relationships between increased TN levels and GDM cannot be established. Serum TN was measured at a single time point; therefore, longitudinal changes throughout pregnancy and postpartum could not be evaluated. In addition, serum samples were stored at −80 °C for variable durations, with some samples stored for up to approximately 26 months before analysis. Although all samples were maintained under identical storage conditions and repeated freeze–thaw cycles were avoided, the potential effect of prolonged and variable storage duration on tetranectin stability cannot be entirely ruled out. We also did not assess TN expression in placental or adipose tissue, which limits mechanistic interpretation. In addition, other adipokines, inflammatory markers, and markers of endothelial dysfunction were not measured; therefore, the relationship between TN and broader inflammatory, vascular, or adipometabolic pathways could not be directly examined. Furthermore, because the proportion of women with GDM was determined by the case–control design rather than the underlying prevalence in the source population, the positive and negative predictive values derived from this sample should not be directly extrapolated to routine clinical populations. Finally, although TN showed moderate discriminatory performance, no independent validation cohort was available to confirm the proposed cut-off value.

## 5. Conclusions

In conclusion, serum TN levels were significantly elevated in women with GDM and remained independently associated with GDM status after adjustment for maternal age, gestational age at sampling, and BMI. Serum TN showed positive correlations with several metabolic parameters; however, after multiple-testing correction, only the associations with fasting glucose and HbA1c remained statistically significant. These correlations should be interpreted cautiously, as they may partly reflect the glycemic separation between the GDM and control groups. Although TN demonstrated moderate discriminatory performance, its potential role should currently be considered exploratory and complementary rather than diagnostic. Larger, longitudinal, multicenter studies are required to validate these findings, determine whether TN provides information independent of glycemic status, and clarify its potential contribution to biomarker-based risk stratification in GDM.

## Figures and Tables

**Table 1:** Baseline maternal and obstetric characteristics of the study groups.

Variables	GDM Group (*n* = 45)	Control Group (*n* = 44)	Effect Size (95% CI)	*p*-Value
Maternal age, years	30.4 ± 6.2	28.0 ± 5.4	0.37 (−0.04–0.80)	0.086 ^1^
Gestational age at sampling, weeks	25.9 ± 1.1	25.8 ± 1.1	0.16 (−0.26–0.58)	0.448 ^1^
Gravida	2.00 [1.00–4.00]	2.00 [1.00–3.00]	0.06 (−0.18–0.29)	0.634 ^2^
Parity	1.00 [0.00–2.00]	1.00 [0.00–1.25]	0.07 (−0.15–0.30)	0.524 ^2^
Abortus	0.00 [0.00–0.00]	0.00 [0.00–0.00]	0.04 (−0.14–0.21)	0.689 ^2^
Body mass index, kg/m^2^	27.7 [25.3–33.7]	27.3 [25.5–30.5]	0.14 (−0.11–0.37)	0.251 ^2^
Gestational weight gain, kg	8.0 ± 4.1	8.4 ± 3.1	−0.13 (−0.65–0.33)	0.590 ^1^
Systolic blood pressure, mmHg	110.0 [110.0–120.0]	110.0 [110.0–120.0]	−0.01 (−0.27–0.25)	0.968 ^2^
Diastolic blood pressure, mmHg	70.0 [60.0–80.0]	70.0 [60.0–80.0]	−0.05 (−0.32–0.21)	0.732 ^2^

Values are presented as mean ± standard deviation or median [interquartile range], depending on the data distribution. ^1^ Independent-samples *t*-test; ^2^ Mann–Whitney U test. Effect sizes are reported as Hedges’ g for normally distributed variables and rank-biserial correlation for non-normally distributed variables, with 95% confidence intervals.

**Table 2:** Glucose metabolism and laboratory parameters.

Variables	GDM Group (*n* = 45)	Control Group (*n* = 44)	*p*-Value
OGTT fasting glucose, mg/dL	96.0 [93.0–103.0]	81.5 [77.0–85.2]	<0.001 ^1^
OGTT 1 h glucose, mg/dL	154.8 ± 40.4	122.5 ± 21.3	<0.001 ^2^
OGTT 2 h glucose, mg/dL	150.0 [120.0–154.0]	95.5 [88.0–111.5]	<0.001 ^1^
Insulin, µIU/mL	11.87 [8.31–15.88]	7.52 [4.45–11.01]	0.003 ^1^
HOMA-IR	2.68 [1.92–3.90]	1.53 [0.95–2.18]	<0.001 ^1^
HbA1c, %	5.38 ± 0.70	4.67 ± 0.42	<0.001 ^2^
Hemoglobin, g/dL	11.5 [10.8–11.9]	11.6 [10.7–11.9]	0.674 ^1^
Hematocrit, %	34.6 ± 3.2	34.0 ± 2.6	0.420 ^2^
WBC/µL	10,800.0 [9500.0–12,500.0]	10,300.0 [9300.0–12,600.0]	0.633 ^1^
Platelet/µL	227,529.41 ± 58,472.08	217,363.64 ± 44,346.52	0.427 ^2^
Total protein, g/dL	6.52 ± 0.48	6.58 ± 0.31	0.558 ^2^
Albumin, g/dL	3.40 [3.30–3.58]	3.30 [3.20–3.40]	0.174 ^1^
Triglyceride, mg/dL	210.50 [184.50–260.75]	199.00 [163.00–242.00]	0.665 ^1^
LDL cholesterol, mg/dL	123.00 [90.25–163.75]	128.00 [110.00–163.00]	0.697 ^1^
HDL cholesterol, mg/dL	62.53 ± 12.30	64.36 ± 14.84	0.583 ^2^
VLDL cholesterol, mg/dL	42.00 [37.00–49.75]	40.00 [33.00–48.00]	0.598 ^1^
BUN, mg/dL	7.00 [5.25–7.75]	7.00 [7.00–8.00]	0.220 ^1^
Total cholesterol, mg/dL	234.50 [203.25–274.25]	235.00 [202.00–278.00]	0.812 ^1^
AST, U/L	16.00 [13.25–18.75]	15.00 [13.00–17.00]	0.259 ^1^
ALT, U/L	13.00 [11.00–15.00]	13.00 [10.00–16.00]	0.546 ^1^
LDH, U/L	191.00 [167.00–209.00]	198.00 [174.00–209.00]	0.935 ^1^
Creatinine, mg/dL	0.60 [0.53–0.60]	0.60 [0.50–0.60]	0.424 ^1^

Values are presented as mean ± standard deviation or median [interquartile range], depending on the data distribution. ^1^ Mann–Whitney U test, ^2^ Independent-samples *t*-test.

**Table 3:** Correlation analysis between serum TN levels and clinical/metabolic parameters.

Parameter	Spearman’s rho	*p*-Value
Maternal age, years	0.084	0.435
Gestational age at sampling, weeks	0.005	0.963
Gravida	−0.033	0.759
Parity	−0.079	0.463
Abortus	0.030	0.783
Systolic blood pressure, mmHg	0.072	0.565
Diastolic blood pressure, mmHg	0.241	0.049
Height	0.037	0.733
Weight, kg	0.270	0.011
Body mass index, kg/m^2^	0.224	0.034
Gestational weight gain, kg	0.176	0.153
Hemoglobin, g/dL	0.053	0.671
Hematocrit, %	0.137	0.270
WBC/µL	0.208	0.092
Platelet/µL	−0.190	0.124
Total protein, g/dL	0.126	0.311
OGTT fasting glucose, mg/dL	0.350	**<0.001**
OGTT 1 h glucose, mg/dL	0.157	0.141
OGTT 2 h glucose, mg/dL	0.277	0.009
Insulin, µIU/mL	0.191	0.082
HOMA-IR	0.246	0.024
HbA1c, %	0.431	**<0.001**
Albumin, g/dL	0.154	0.214
Triglyceride, mg/dL	0.243	0.047
LDL cholesterol, mg/dL	0.034	0.785
HDL cholesterol, mg/dL	−0.121	0.327
VLDL cholesterol, mg/dL	0.236	0.054
BUN, mg/dL	0.203	0.100
Total cholesterol, mg/dL	0.035	0.779
AST, U/L	−0.147	0.234
ALT, U/L	0.154	0.214
LDH, U/L	−0.047	0.706
Creatinine, mg/dL	0.047	0.706
Gestational age at delivery, weeks	0.006	0.956

Correlation analyses were performed using Spearman’s rank correlation test. To account for multiple comparisons, a Bonferroni-corrected significance threshold of *p* < 0.00147 was applied for the 34 correlations tested. Bold *p*-values indicate associations that remained statistically significant after Bonferroni correction.

**Table 4:** Binary logistic regression analysis for the association between serum TN levels and GDM.

Model	Variable	OR	95% CI	*p*-Value
Crude model	Tetranectin, per 10 ng/mL increase	1.17	1.07–1.28	0.001
Adjusted model	Tetranectin, per 10 ng/mL increase	1.17	1.06–1.28	0.001
Adjusted model	Maternal age, years	1.06	0.98–1.16	0.154
Adjusted model	Gestational age at sampling, weeks	1.18	0.77–1.81	0.435
Adjusted model	Body mass index, kg/m^2^	1.00	0.91–1.11	0.963

Binary logistic regression analysis was performed with GDM status as the dependent variable. Serum tetranectin was entered into the model as a continuous variable, expressed per 10-ng/mL increase. The adjusted model included maternal age, gestational age at sampling, and body mass index as clinically relevant potential confounders. OR: odds ratio; CI: confidence interval.

## Data Availability

The datasets are not publicly available. The de-identified data are available upon request from the corresponding author due to privacy, ethical, and legal restrictions that protect patient confidentiality.

## Abbreviations

The following abbreviations are used in this manuscript:

ALT	Alanine aminotransferase
AST	Aspartate aminotransferase
AUC	Area under the curve
BMI	Body mass index
BUN	Blood urea nitrogen
CI	Confidence interval
CLEC3B	C-type lectin domain family 3 member B
ELISA	Enzyme-linked immunosorbent assay
GDM	Gestational diabetes mellitus
HbA1c	Glycated hemoglobin
HDL	High-density lipoprotein
HOMA-IR	Homeostatic Model Assessment for Insulin Resistance
HRP	Horseradish peroxidase
LDH	Lactate dehydrogenase
LDL	Low-density lipoprotein
MAPK	Mitogen-activated protein kinase
OCT4	Octamer-binding transcription factor 4
OGTT	Oral glucose tolerance test
OR	Odds ratio
ROC	Receiver operating characteristic
TN	Tetranectin
TXNIP	Thioredoxin-interacting protein
VLDL	Very-low-density lipoprotein
WBC	White blood cell
